# Targeting lung cancer cells with MUC1 aptamer-functionalized PLA-PEG nanocarriers

**DOI:** 10.1038/s41598-022-08759-z

**Published:** 2022-03-18

**Authors:** Shima Shahrad, Mohammad Rajabi, Hamidreza Javadi, Ali Akbar Karimi Zarchi, Mohammad Hasan Darvishi

**Affiliations:** 1 Department of Materials Engineering, Babol Noshirvani University of Technology, Shariati Ave., 47148-71167 Babol, Iran; 2 Nanobiotechnology Research Center, Baqiyatallah University of Medical Sciences, Tehran, Iran

**Keywords:** Biotechnology, Cancer

## Abstract

MUC1 aptamer-functionalized PLA-PEG nanocarriers at various w/w ratios (polymer to doxorubicin weight ratio) were prepared by a double emulsion method. Physiochemical properties, encapsulation efficiency (EE), loading content (LC) and in vitro release kinetics of DOX were assessed. Furthermore, cytotoxicity and antitumor activity of prepared PLA-PEG-Apt/DOX NPs at w/w ratio 10:1 were evaluated by MTT assay and flow cytometry against MUC1-overexpressing A-549 cell line. Targeted nanocarriers (PLA-PEG-Apt/DOX NPs at w/w ratio 10:1) induced higher apoptosis rate (36.3 ± 3.44%) for 24 h in MUC1 positive A-549 cancer cells in compare to non-targeted form (PLA-PEG/DOX NPs at w/w ratio 10:1, 11.37 ± 1.65%) and free DOX (4.35 ± 0.81%). In other word, the percentage of cell death in A-549 lung cancer cells treated with PLA-PEG-Apt/DOX NPs at w/w ratio 10:1 is 3.19 and 8.34 fold higher than in non-targeted form and Free DOX treated cancer cells, respectively. Therefore, PLA-PEG-Apt/DOX NPs might be considered a promising drug delivery system for targeted drug delivery towards MUC1-overexpressing tumors cells.

## Introduction

Cancer is among the leading cause of death worldwide and responsible for nearly 10 million deaths in 2020^1^. Among various cancer types, lung cancer, with approximately 1.8 million deaths (18%), is considered to be the most common cause of cancer death^2,3^. Non-small cell lung cancer (NSCLC) accounts for about 80–85% of total cases of lung cancer and remains the most common malignancy of lung cancer ^4,5^. Chemotherapy as a type of cancer treatment suffer from limitations such as lack of specificity and selectivity toward cancer cells beside to systemic toxicity, severe side effects and drug resistance^6,7^. In order to pass over these obstacles, nanocarrier based formulations have been developed to improve the therapeutic efficacy while reducing side​-effects of therapeutic agents^8–11^. Poly (lactide) (PLA) NPs have been widely used for controlled release of various chemotherapeutic drugs due to their desirable properties such as good biocompatibility, biodegradability, nontoxicity and sustained release features^12–15^. In order to protect PLA NPs from phagocytosis by the reticuloendothelial system (RES), improve the systemic retention and prolong blood circulation, they have been modified with poly (ethylene glycol) (PEG)^12,14,16,17^.

Doxorubicin (DOX) is one of the effective and widely used chemotherapeutic anticancer drugs which is used in the treatment of various solid tumors including breast, ovarian, lung and prostatic cancer^18–21^. systemic administration of DOX leads to severe adverse side effects including cardio toxicity and heart failure^22,23^. DOX loaded NPs improve therapeutic efficacy by increasing its bioavailability and reducing its severe side effects and toxicity^24,25^.

Active tumor targeting can be achieved by conjugating different tissue-specific groups and ligands including antibodies, peptides, aptamers and vitamins to nanoparticles surface^26–32^. Therefore, targeted drug delivery using targeting ligands have been developed in order to target overexpressed receptors in tumor tissue due to their advantages such as selective delivery of drug loaded nanoparticles, enhancing drug accumulation, and increasing cellular uptake at tumor site^33–37^. Aptamers are short, single stranded oligonucleotides DNA or RNA with particular three dimensional (3D) structure that bind to targets with high affinity and specificity^38–40^. Aptamers offer significant advantages, including small size followed by rapid tissue penetration, ease of synthesis and chemical modification, Low toxicity and immunogenicity, stability, and long shelf-life^41–44^. Such properties make aptamers a suitable alternative for monoclonal antibodies, peptide and protein ligands^36,45^. Therefore, aptamers have been widely used as smart binding ligands in many biomedical approaches such as diagnostic assays, biomarker discovery, and targeted drug delivery^39,45,46^. Mucin 1 (MUC1) is a heterodimeric protein formed by two subunits that is highly overexpressed (until tenfold) on the surface of cancer cells in most malignant tumors including ovarian, lung, pancreatic, prostate, and breast cancers^47–50^. Recently, MUC1 aptamers (Apt) have been developed to specifically target MUC1 protein for enhanced drug delivery in cancer treatment^51^. MUC1 aptamer S2.2, is a 25-nucleotide small single stranded DNA with unique 3-dimensional structures which can selectively binds to MUC1-expressing cancer cells such as A-549 (human alveolar basal epithelial cells) with high affinity and specificity^27,48,52–54^.

We have developed a novel tumor targeted delivery system based on aptamer targeted nanoparticles (PLA-PEG-Apt/DOX NPs) for efficient anticancer drug delivery to lung cancer cells. In this work, MUC1 aptamer functionalized PLA-PEG nanocarrier is used for targeted delivery of doxorubicin to MUC1-positive lung cancer cells.

The physicochemical properties of PLA-PEG-Apt/DOX NPs including size and zeta potential were investigated. The encapsulation efficiency, loading content and release profile of the PLA-PEG-Apt/DOX NPs were evaluated in vitro condition. The conjugation of MUC1 aptamer to PLA-PEG/DOX NPs was confirmed. The morphology of PLA-PEG-Apt/DOX NPs was studied by SEM. Furthermore, the antitumor efficacy of PLA-PEG-Apt/DOX NPs was evaluated by in vitro cytotoxicity and cellular apoptosis study in MUC1-overexpressing model A-549 cell line.

## Materials and methods

### Materials

d,l-Lactic and the polyethylene glycol with a carboxylic acid functional group (OH-PEG_3400_-COOH) was obtained from Sigma-Aldrich (St. Louis, MO, USA). Doxorubicin (DOX) was purchased from RPG Life Sciences limited (Mumbai, India). 5′ amino-modified MUC1 DNA Aptamer S2.2 with sequence of 5′ GCAGTTGATCCTTTGGATACCCTGG 3′ was synthesized by Metabion International AG (Planegg, Germany). RPMI 1640 medium, fetal bovine serum (FBS) and 0.25% trypsin were purchased from Gibco BRL (Carlsbad, CA, USA). A-549 cell line was obtained from the National Cell Bank of Iran (NCBI). All other chemicals were obtained from Sigma- Aldrich (St. Louis, MO, USA).

### Synthesis of PLA-PEG–COOH

**Figure 1:**
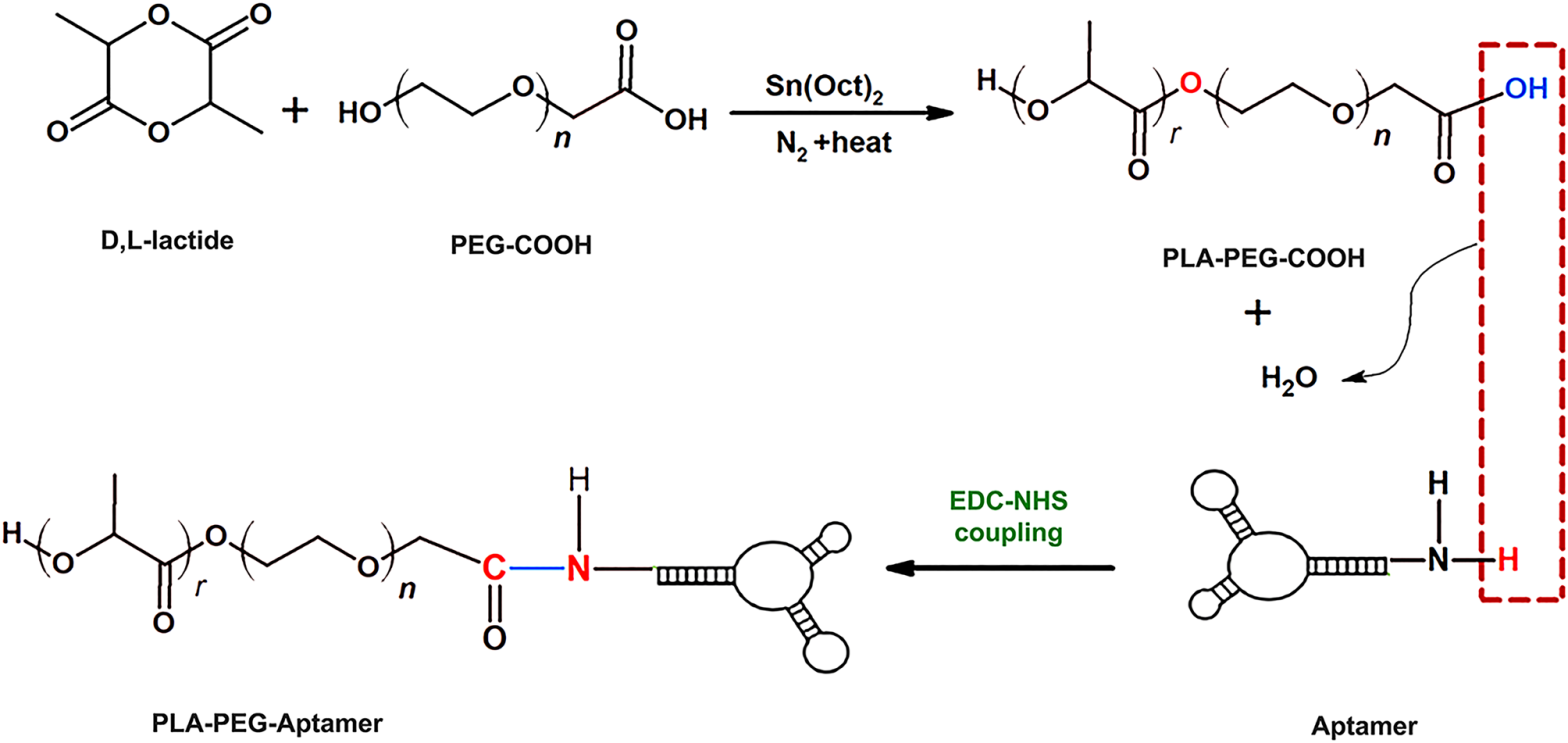
Schematic illustration of synthesizing PLA-PEG–COOH and preparing aptamer conjugated nanoparticles.

The copolymers of PLA-PEG–COOH were synthesized by ring-opening polymerization using previously reported with some modifications (Fig. 1)^55^. Glassware were salinized by rinsing with a 5% methyl trichlorosilane solution in toluene, followed by rinsing with acetone, and left overnight to dry at 130 °C. Carboxylated PEG (0.02 g) and lactide monomers (0.1 g) were added to a round bottom flask and dissolved in 7 ml dried toluene and stannous octoate as a catalyst was then added to the solution and the reaction was carried out 6 h at 120 °C under nitrogen. The organic solvent was evaporated under vacuum condition by rotary evaporator. Unreacted lactide monomers were hydrolyzed by adding cold water and PLA-PEG–COOH copolymers crystalized by adding acetonitrile and chilled methanol under stirrer in room temperature. The mixture was then centrifuged for 30 min at 10,000 rpm. The resulting PLA-PEG–COOH was characterized by ^1^H-NMR (500 MHz spectrometer, Bruker Ac 500, Germany) and FTIR spectrophotometry (Nicolet 550 A, USA).

### Preparation of PLA-PEG/DOX NPs

The PLA-PEG/DOX NPs were prepared by a double emulsion method^18^. In detail, 500 μl DOX (2 mg/ml) was added drop-wise into 500 μl of dichloromethane containing 5, 10 and 20 mg PLA-PEG copolymer (PLA-PEG/DOX at various w/w ratios 5:1, 10:1, 20:1), and then the two-phase mixture was emulsified by sonication for 1 min (70 w) in an ice bath. Subsequently, 2 ml of PVA solution (3% w/v) was added and sonicated for 1 min to form a w/o/w double emulsion and stirred further for 5 min. The resulting double emulsion was diluted into 10 ml of a 0.3% (w/v) PVA aqueous solution under stirring for 5 min, and then the organic solvent was evaporated by rotary evaporator under vacuum. The resulting nanoparticles were centrifuged at 15,000 rpm for 30 min, washed with deionized water and lyophilized.

### Conjugation of MUC1 aptamers to nanoparticles

The conjugation of MUC1 aptamers with 5′ terminal amino group modification to PLA-PEG/DOX NPs (w/w ratio 10:1) was accomplished via crosslinking of –COOH group of PEG–COOH and –NH_2_ group of MUC1 aptamer (Fig. 1)^56^. Briefly, 100 μl of PLA-PEG/DOX NPs suspension (~ 10 μg/μl in DNase RNase-free water) was incubated with 100 μl of 400 mmol/l 1-(3-dimethylaminopropyl)-3-ethylcarbodimide hydrochloride (EDC) and 100 μl of 100 mmol/l *N*-hydroxysuccinimide (NHS) for 30 min at room temperature with gentle stirring. Then the NHS-activated particles were covalently linked to 10 μl of 5′-NH_2_-modified MUC1 aptamer (1 μg/μl in DNase RNase-free water) for 3 h at room temperature. The resulting aptamer conjugated nanoparticles were separated from unconjugated aptamers by centrifugation, washed and resuspended in DNase RNase-free. The attachment of aptamers to PLA-PEG NPs was confirmed by agarose gel electrophoresis.

### Characterization of PLA-PEG-Apt/DOX NPs

The particle size, polydispersity index (PDI) and zeta potential analyses of the PLA-PEG-Apt/DOX NPs were evaluated by DLS (Malvern Zeta sizer 3000HS, Malvern, UK). The morphological characteristics of the PLA-PEG-Apt/DOX NPs were examined by scanning electron microscopy (SEM) (SEM; TESCAN MIRA3, Prague, Czechoslovakia). The confirmation of attachment of MUC1 Aptamers to PLA-PEG NPs was studied by agarose gel electrophoresis. For this purpose, the samples including PLA-PEG-Apt/DOX NPs, PLA-PEG, the mixture of PLA-PEG and aptamer solution, and free MUC1 aptamer were separately loaded on 2% agarose gel. Then the electrophoresis was performed at 90 V for 30 min in Tris–borate-EDTA buffer (1 × TBE, PH 8.3). The aptamer was visualized after incubating the gel in DNA safe stains under an ultraviolet trans-illuminator (Syngene, USA).

### Encapsulation efficiency and drug loading

The Dox entrapment efficiency and loading content of NPs were determined by UV spectrophotometry. Thus, the freeze-dried nanoparticles were dissolved in DMSO, and then the supernatant was collected by centrifugation. The concentration of DOX in the supernatant was monitored using a Visible–UV spectrophotometer (Varian, Cary 100 UV/Vis Spectrophotometer, and USA) at 480 nm. The encapsulation efficiency (EE) and drug loading of DOX (LC) in the NPs were calculated according to following formulas^18^:

1 $${\text{Drug}}\;{\text{loading}}\;{\text{content}}\left( \% \right) = \frac{{{\text{The}}\;{\text{weight}}\;{\text{of}}\;{\text{DOX}}\;{\text{in}}\;{\text{the}}\;{\text{nanoparticles}}}}{{{\text{The}}\;{\text{gross}}\;{\text{weight}}\;{\text{of}}\;{\text{nanoparticles}}}} \times 100$$

2 $${\text{Encapsulation }}\;{\text{efficiency}}\left( \% \right) = \frac{{{\text{The}}\;{\text{weight}}\;{\text{of}}\;{\text{DOX}}\;{\text{in}}\;{\text{the}}\;{\text{nanoparticles}}}}{{{\text{The}}\;{\text{total}}\;{\text{weight}}\;{\text{of}}\;{\text{the}}\;{\text{feeding}}\;{\text{DOX}}}} \times 100$$

### In vitro release study

Drug (Dox) release from NPs was studied using a dialysis method. For this, freeze-dried PLA-PEG-Apt/DOX NPs were placed into a dialysis bag (molecular weight cutoff 12 kDa), and the bag was subsequently immersed in a flask containing 30 ml of phosphate-buffered saline (PBS, pH 7.4). The release medium was then incubated in a shaking water bath maintained at 37 °C and 100 rpm. The samples were withdrawn from the medium at time intervals of 0.5, 1, 2, 4, 6, 8, 20, 24, 48, 72 and 96 h. After each sampling an equal volume of fresh PBS was added to maintain a sink condition. The released DOX was measured at 480 nm by a UV/Vis spectrophotometer.

### Cell line experiments

#### Cell culture

A-549 cell line (human alveolar basal epithelial cells) was purchased from national Cell Bank of Iran (Pasteur Institute, Tehran, Iran). The cells were cultured in RPMI 1640 medium (Gibco, BRL, USA), supplemented with 10% FBS (Gibco), streptomycin (100 ng/ml), and penicillin (100 U/mL) at 37 °C in 5% CO_2_ humidified incubator.

### In-vitro cytotoxicity

The cytotoxicity of PLA-PEG-Apt/DOX NPs was analyzed in-vitro via MTT assay. Briefly, the cells were seeded into 96 well plates at the density of 1 × 10^4^ cells/well and incubated for 24 h to reach 70–80% confluences. Then, the cells were treated with free DOX, PLA-PEG, PLA-PEG/DOX NPs (w/w ratio 10:1), and PLA-PEG-Apt/DOX NPs (w/w ratio 10:1) and incubated for 24 h at 37 °C. The untreated cells were used as controls. After incubation time, supernatant was removed and the cells were washed with PBS. Subsequently, 10 μl of MTT solution (5 mg/ml) was added into the wells and the plate was incubated for 4 h in 5% CO_2_ at 37 °C. Afterwards, the medium was replaced with 100 μl DMSO and mixed with shaking for 20 min to dissolve the formazan crystals. Finally, the absorbance of the wells was obtained at 570 nm using the microplate reader (Stat Fax 2100, Block Scientific, and USA). The cell viability rate was calculated by the following equation^14^:

3 $${\text{Cell}}\;{\text{viability}}\left( \% \right) = \frac{{{\text{Absorption}}\;{\text{value}}\;{\text{of}}\;{\text{treatment}}\;{\text{group}}}}{{{\text{Absorption}}\;{\text{value}}\;{\text{of}}\;{\text{control}}\;{\text{group}}}} \times 100$$

### Flow cytometry

The Targeting property and cellular apoptosis of PLA-PEG-Apt/NPs was determined in A-549 cells by flow cytometry analysis (FCM) using Annexin V-FITC apoptosis detection kit. For this purpose, A-549 cells were seeded into 12-well plates with 1 ml of 5 × 10^4^ cells per well in growth medium for 24 h at 37 °C. Then, the medium was removed and the cells were treated with free DOX, PLA-PEG NPs, PLA-PEG/DOX NPs (w/w ratio 10:1), PLA-PEG-Apt NPs, and PLA-PEG-Apt/DOX NPs (w/w ratio 10:1) suspended in 1 ml fresh medium and incubated for 24 h in 5% CO_2_ at 37 °C. After incubation time and removal of the culture medium, the cells were trypsinized, washed three times with PBS and collected by centrifugation. Then, the cells were re-suspended in 100 μl of binding buffer, 5 μl of FITC-labelled annexin V was added, and incubated for 20 min at room temperature. Finally, the cells were analysed by FACS caliber flow cytometer (Becton Dickinson Immunocytometry Systems, San Jose, CA). Moreover, cellular internalization of PLA-PEG-Apt/DOX NPs was observed with a fluorescence microscope (Leica Microsystems Inc., Buffalo Grove, IL).

### Statistical analysis

Statistical analysis of the experiments such as MTT assay and apoptosis study was performed using GraphPad Prism version 9 (GraphPad Software, USA). All data are reported as mean ± SD. Statistical comparisons was calculated using Tukey’s multiple comparisons test and one-way ANOVA analysis at P values ≤ 0.05 as significance level.

### Ethics approval

The manuscript does not contain clinical studies or patient data.

## Results and discussion

### Synthesis and characterization of PLA-PEG–COOH

**Figure 2:**
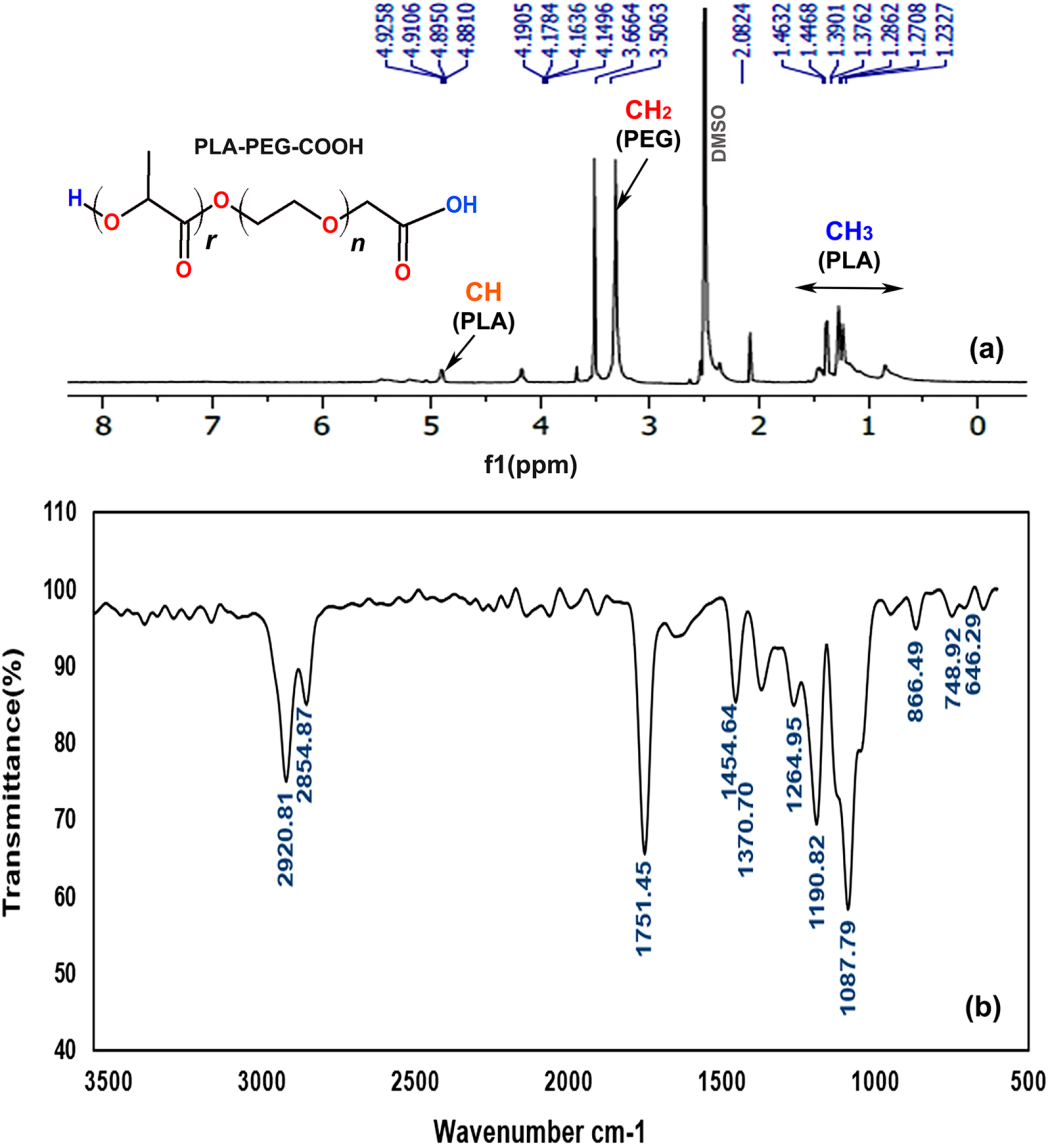
(**a**) H NMR^1^ spectrum (500 MHz, DMSO), and (**b**) FTIR spectrum of PLA-PEG–COOH.

The PLA-PEG–COOH copolymer was synthesized via the ring-opening polymerization of the lactide in the presence of carboxylated PEG^55,56^. The H-NMR^1^ spectrum of PLA-PEG–COOH copolymer is shown in Fig. 2a. The strong peak at 3.5 ppm belongs to the CH_2_ protons of PEG. The small peaks around 1.2–1.4 ppm and 4.9 ppm correspond to the CH_3_ and CH protons of PLA, respectively. The H-NMR1 spectrum indicates the ring opening polymerization of lactide and confirms the formation of block copolymer^56^. The FTIR spectrum of PLA-PEG–COOH copolymer is shown in Fig. 2b. The copolymer presented peaks for stretching C–H bonds of alkyl groups at 2854.87–2920.81/cm, ether C–O–C stretch at 1087.89/cm and C=O bond of COOH at 1454.64/cm associated with the PEG block of PLA-PEG–COOH. Besides, the characteristic sharp C=O stretch peak at 1751.45/cm related to the PLA block of PLA-PEG–COOH which indicates that carboxylic acid groups of PLA successfully reacted with OH group of PEG^55^. Thus, the FTIR spectrum strongly confirms the synthesis of PLA-PEG–COOH copolymer.

### Preparation and characterization of PLA-PEG-Apt/DOX NPs

The PLA-PEG-Apt/DOX nanoparticles were prepared by a double emulsion (W/O/W) method and EDC/NHS technique^18,25,56,61^. Physiochemical properties of prepared NPs including particle size, polydispersity index (PDI), and zeta potential were evaluated by dynamic light scattering (DLS) as shown in Table 1. The average size of PLA-PEG-Apt/DOX NPs at various w/w ratios (PLA-PEG: DOX) 5:1, 10:1, and 20:1 (w/w) ranged between 134.16 and 178.18 nm. As shown in Table 1, increasing the w/w ratio of PLA-PEG: DOX from 5:1 to 20:1 (w/w) resulted in constant increase in the sizes of the nanoparticles. That is because, increasing the polymer concentration is associated with enhancing the viscosity of organic phase which restricts diffusion of organic solvent in aqueous phase and subsequently Leads to the formation of larger nanoparticles^57^. The zeta potential of PLA-PEG-Apt/DOX NPs at various ratios were in the range of − 18.16 (w/w ratio 5:1) to − 24.95 mV (w/w ratio 20:1). It has been shown that increasing the w/w ratio from 5:1 to 20:1 leads to increase in nanoparticles surface charges (Table 1). The sufficient amounts of negative charge of prepared nanoparticles lead to repulsion between particles and prevent their aggregation. Furthermore, the PDI of nanoparticles is also increased constantly with increase in w/w ratio from 5:1 to 20:1 (w/w). The particle size and surface charge are the key factors in the development of nanoparticles-based delivery systems due to their impact on half-life of the NPs in blood circulation, nanoparticle–cell interactions, and their cellular uptake^14^.

**Table 1:** Size, zeta potential and PDI of PLA-PEG-Apt/DOX NPs.

Sample	Z-average ± SD (nm)	PDI ± SD	Z potential ± SD (mV)
PLA-PEG-Apt/DOX NPs (5:1)	134.16 ± 4.77	0.263 ± 0.057	− 18.16 ± 0.61
PLA-PEG-Apt/DOX NPs (10:1)	157.66 ± 5.72	0.274 ± 0.125	− 20.03 ± 1.96
PLA-PEG-Apt/DOX NPs (20:1)	178.18 ± 5.28	0.379 ± 0.098	− 24.95 ± 1.08

**Figure 3:**
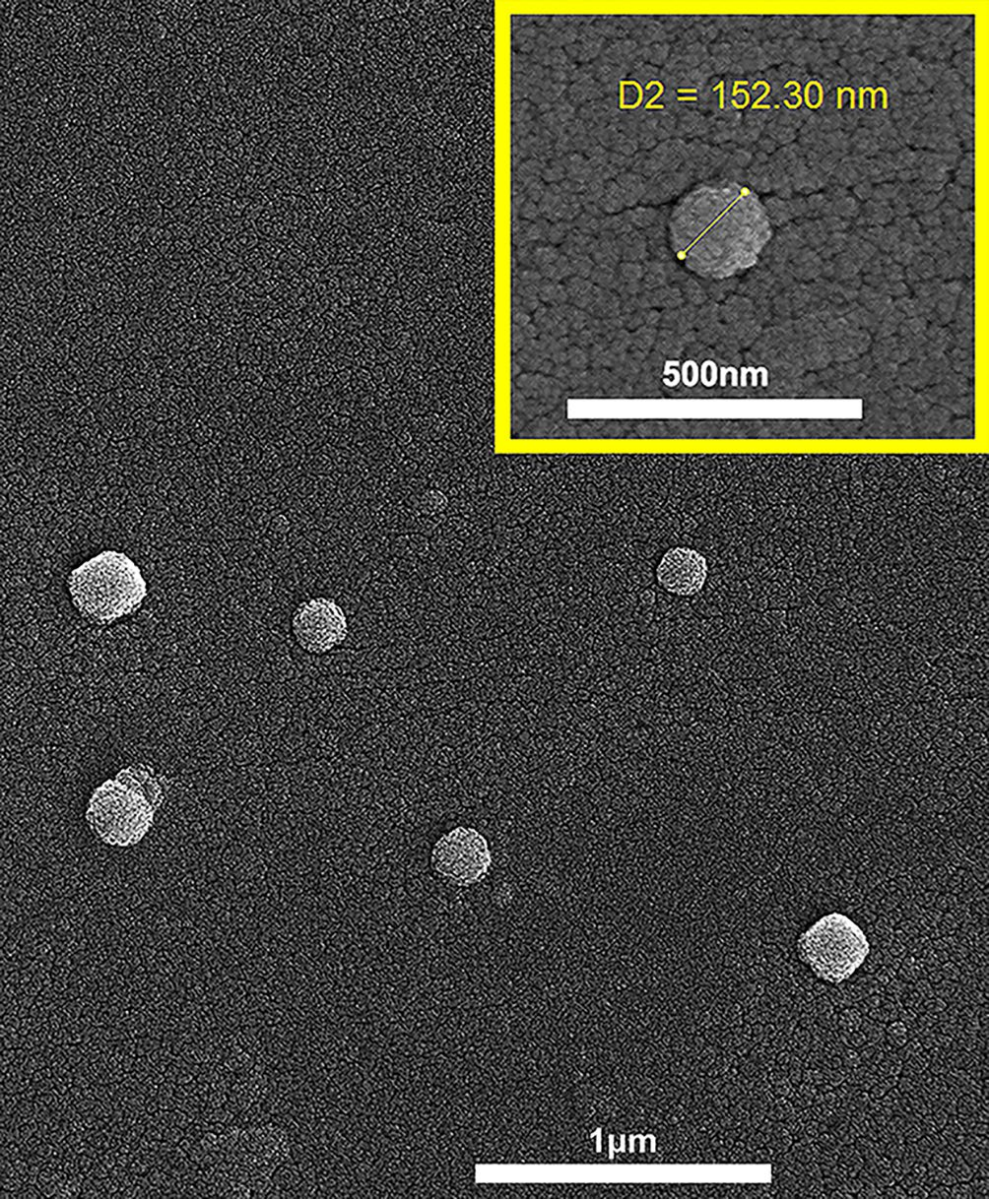
SEM image of PLA-PEG-Apt/DOX NPs (at w/w ratio 10:1).

The morphology of the PLA-PEG-Apt/DOX NPs was characterized using scanning electron microscopy (SEM) and the result presented in Fig. 3 which showed smooth nanoparticles with spherical morphology.

**Figure 4:**
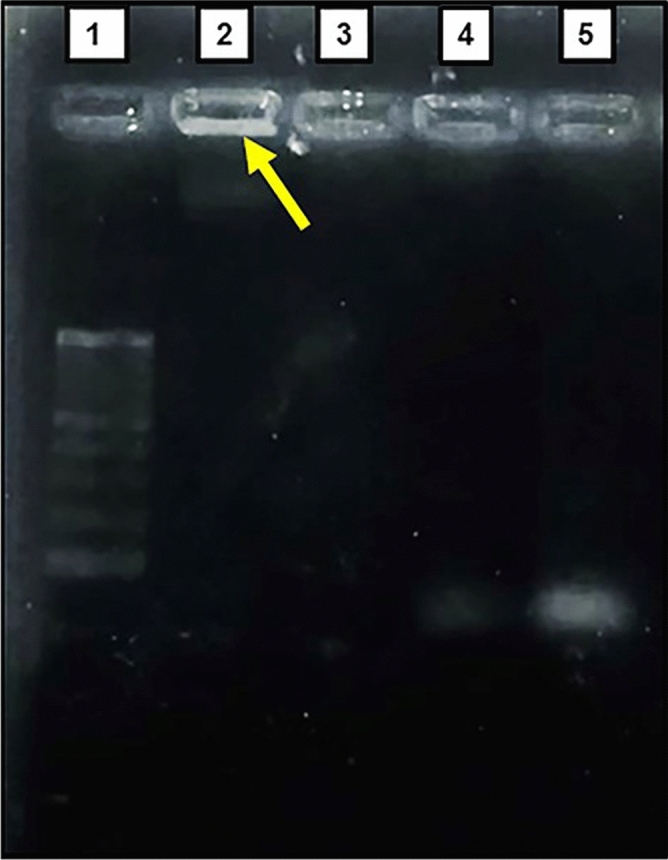
Confirmation of NPs-aptamer conjugation. 1: ladder (100 bp), 2: PLA-PEG-Apt/DOX NPs, 3: PLA-PEG, 4: mixture of aptamers and PLA-PEG, 5: free aptamer (Apt). Original gel is presented in Supplementary Fig. 4.

The conjugation of MUC1 aptamers to nanoparticles was verified using agarose gel electrophoresis. As shown in Fig. 4, four samples were tested to verify conjugation. Aptamers movement through the pores of agarose gel matrix have produced a sharp band on the gel (lane5), while PLA-PEG-Apt/DOX NPs could not move through gel and stayed in the well or moved just a little distance due to their large particle size and showed a band which indicating the presence of aptamers in the well (lane 2). Therefore, illuminated well will be a signal for aptamer conjugated nanoparticles and band through the gel will be a signal for free aptamer. There is a detectable short band on the gel corresponding to the mixture of aptamers and PLA-PEG (lane 4) indicating the presence of unconjugated aptamers and non-covalent conjugation of aptamers and PLA-PEG. No band was detected for PLA-PEG alone (lane 3)^58–60^.

### Encapsulation efficiency and drug loading

In this study, PLA-PEG-Apt/DOX NPs were prepared by a double emulsion method in the presence of DOX and PLA-PEG copolymer. The drug loading and encapsulation characterizations of prepared NPs were summarized in Table 2. Results suggested that w/w ratio of PLA-PEG and DOX strongly influence the encapsulation efficiency (EE) and drug content (LC). For all the fabricated samples, the encapsulation efficiency increased greatly as the w/w ratio of PLA-PEG and DOX increased. As can be seen in Table 2, the EE of PLA-PEG-Apt/DOX NPs at 5:1 w/w ratio was only 16.47 ± 0.2, whereas at 20:1 w/w ratio, the encapsulation efficiency increased to 56.48 ± 2.75. This is in agreement with the works of Li et al. and Ghasemi et al.^18,57^, which presented that the more amount of polymer content led to higher encapsulation efficiency. This is due to the reason that, increasing the polymer concentrations lead to stronger shielding effect of polymer chain on drug which prevents drug from diffusion into polymer solution. On the other hand, it has been indicated that the drug loading content significantly reduced with the addition of w/w ratio of copolymer and drug. The DOX loading efficiency for 5:1 w/w ratio was 6.72 ± 0.5. However, in 20:1 w/w ratio, the drug loading content decreased to 2.45 ± 0.23 (Table 2).

**Table 2:** Encapsulation efficiency and drug loading of PLA-PEG-Apt/DOX NPs.

Sample	Encapsulation efficiency ± SD (%)	Drug loading content ± SD (%)
PLA-PEG-Apt/DOX NPs (5:1)	16.47 ± 0.2	6.72 ± 0.5
PLA-PEG-Apt/DOX NPs (10:1)	44.55 ± 1.61	5.71 ± 0.28
PLA-PEG-Apt/DOX NPs (20:1)	56.48 ± 2.75	2.45 ± 0.23

### In-vitro drug release study

**Figure 5:**
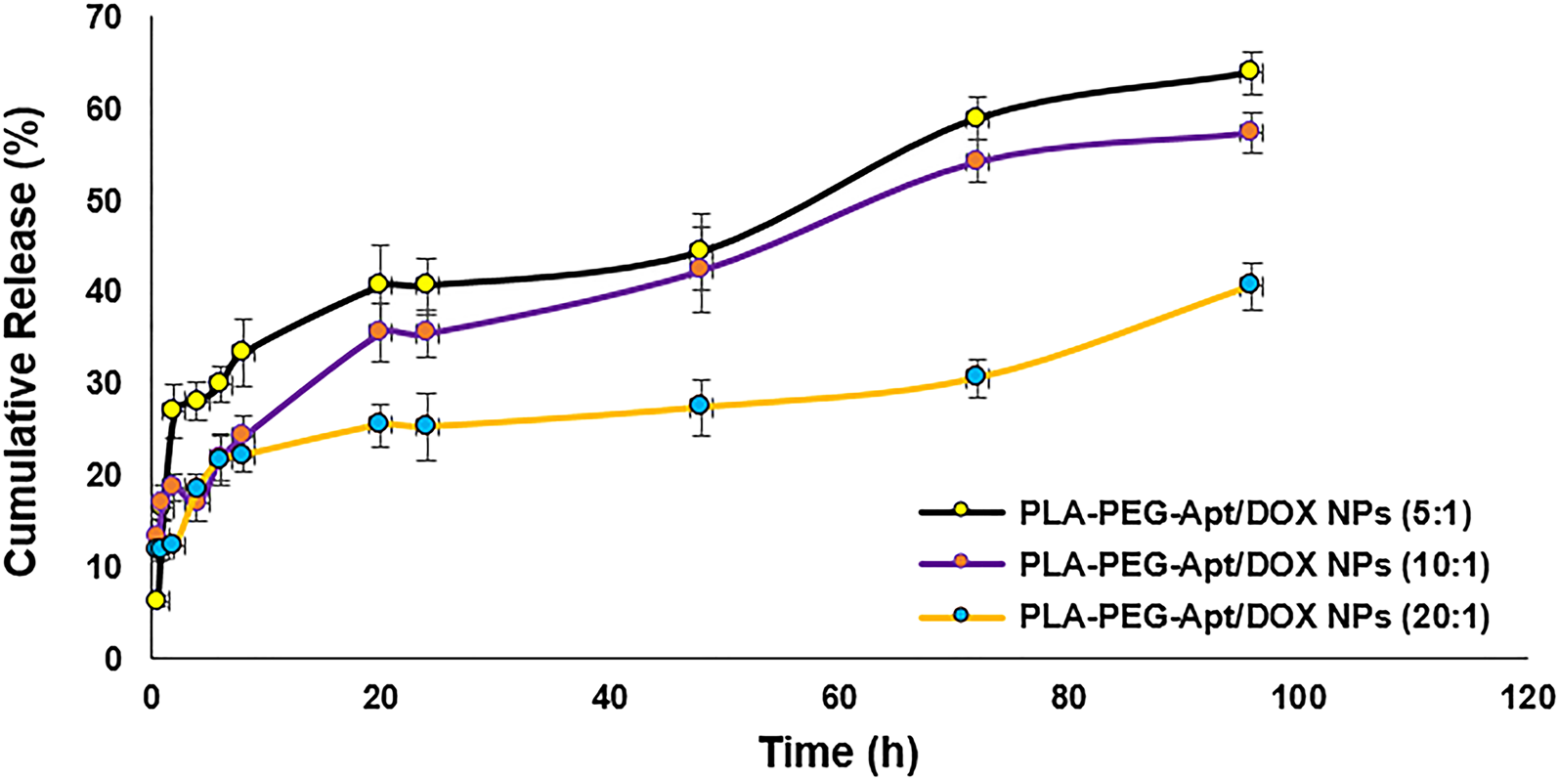
In-vitro release of PLA-PEG-Apt/DOX NPs at various w/w ratios.

In order to investigate the potential of the prepared nanoparticles as carriers of DOX, in vitro release behavior of DOX-loaded nanoparticles was evaluated for 96 h under physiological conditions (pH 7.4 and temperature of 37 °C). The release profile of PLA-PEG-Apt/DOX NPs with 5:1, 10:1, and 20:1 w/w ratios (PLA-PEG: DOX) is illustrated in Fig. 5. PLA-PEG-Apt/DOX NPs at 5:1 w/w ratio expressed sustained release of drug for 96 h. However, a small burst release (about 26% of the total loaded drug) observed in the first 2 h for the nanoparticles with 5:1 w/w ratio, and then followed by a slow drug release. About 63% of the total drug loaded in PLA-PEG-Apt/DOX NPs at 5:1 w/w ratio was gradually released within 96 h. Whereas, PLA-PEG-Apt/DOX NPs at 10:1 and 20:1 w/w ratio showed 57% and 40% release of the total DOX following by a controlled slow release up to 96 h, respectively. It has been declared that drug molecules diffusion and polymer matrix degradation are two mechanisms, which could influence the drug release^61,62^. Furthermore, the drug molecules which are close to the surface of the nanoparticles dispersing rapidly from matrix into buffer and lead to burst release in the first few hours. Therefore, most DOX in 10:1 and 20:1 w/w ratio were far from the surface of the nanoparticles by increasing the polymer concentration. For this reason, there was no obvious burst release of drug for such nanoparticles^14^. In addition, higher polymer concentrations could form the thicker polymer wall, which effectively prevented DOX from releasing in buffer. Due to this fact, PLA-PEG-Apt/DOX NPs at 20:1 w/w ratio showed much less drug release compared to nanoparticles with 5:1 and 10:1 w/w ratio. Based on these results, it can be concluded that PLA-PEG-Apt/DOX NPs at 10:1 w/w ratio with no obvious burst release and 57% controlled release of the total drug during 96 h could be a good candidate for DOX carriers.

### In-vitro cytotoxicity

**Figure 6:**
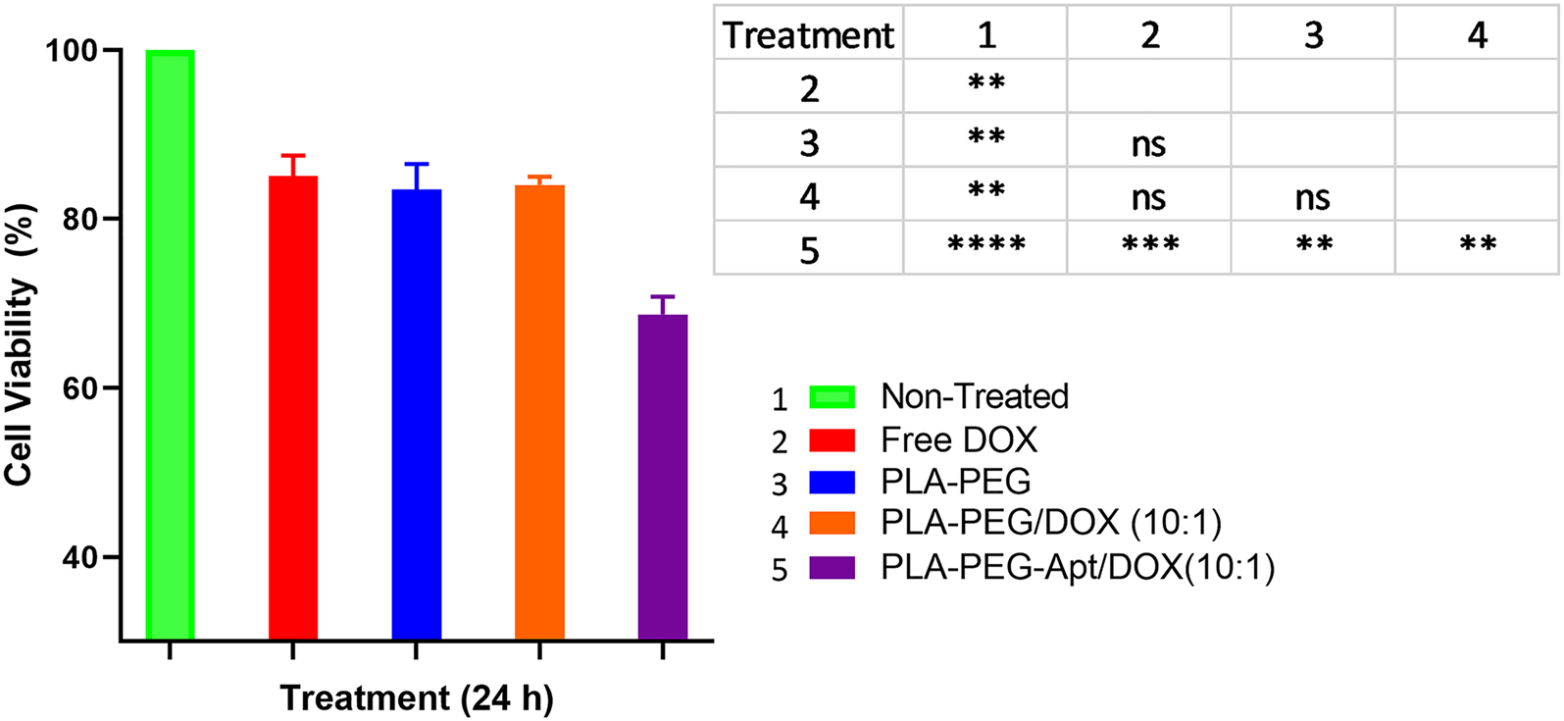
In-vitro cytotoxicity evaluation of DOX, PLA-PEG, PLA-PEG/DOX NPs and PLE-PEG-Apt/DOX NPs in A549 cancer cells at 24 h (P-value: ns is Not-significance (≥ 0.05)., * is significant between 0.01 and 0.05, ** is significant between 0.001 and 0.01, *** is significant between 0.0001 and 0.001, and ****significant < 0.0001).

In-vitro cytotoxicity of different samples such as free DOX, PLA-PEG, PLA-PEG/DOX NPs (w/w ratio 10:1), and PLA-PEG-Apt/DOX NPs (w/w ratio 10:1) was evaluated by MTT assay using A-549 cells (Fig. 6). As shown in Fig. 6, free DOX with cell viability 85.06 ± 2.45%, PLA-PEG with 83.45 ± 3.06%, and PLA-PEG/DOX NPs at w/w ratio 10:1 with 84.03 ± 0.94% showed little cytotoxicity for A-549 cells at 24 h. However, the most cytotoxicity was caused by PLA-PEG-Apt/DOX NPs at w/w ratio 10:1 with cell viability 68.68 ± 2.11% at 24. The results suggesting that PLA-PEG-Apt/DOX NPs at w/w ratio 10:1 produced a more potent cytotoxicity than PLA-PEG/DOX NPs at w/w ratio 10:1. Furthermore, statistically significant differences were observed in cell viability between PLA-PEG-Apt/DOX NPs at w/w ratio 10:1 and other formulations (P < 0.05), while no significant difference was found between the other formulations (Fig. 6). This is because of MUC1 aptamer selectively enhanced the uptake of PLA-PEG-Apt/DOX NPs into A-549 cells and subsequently increased cell toxicity^27,48^. Thus, MUC1 aptamer may selectively increase the delivery of DOX encapsulated in PLA-PEG nanocarriers to A-549 MUC1-positive cancer cells.

### The cellular apoptosis study

**Figure 7:**
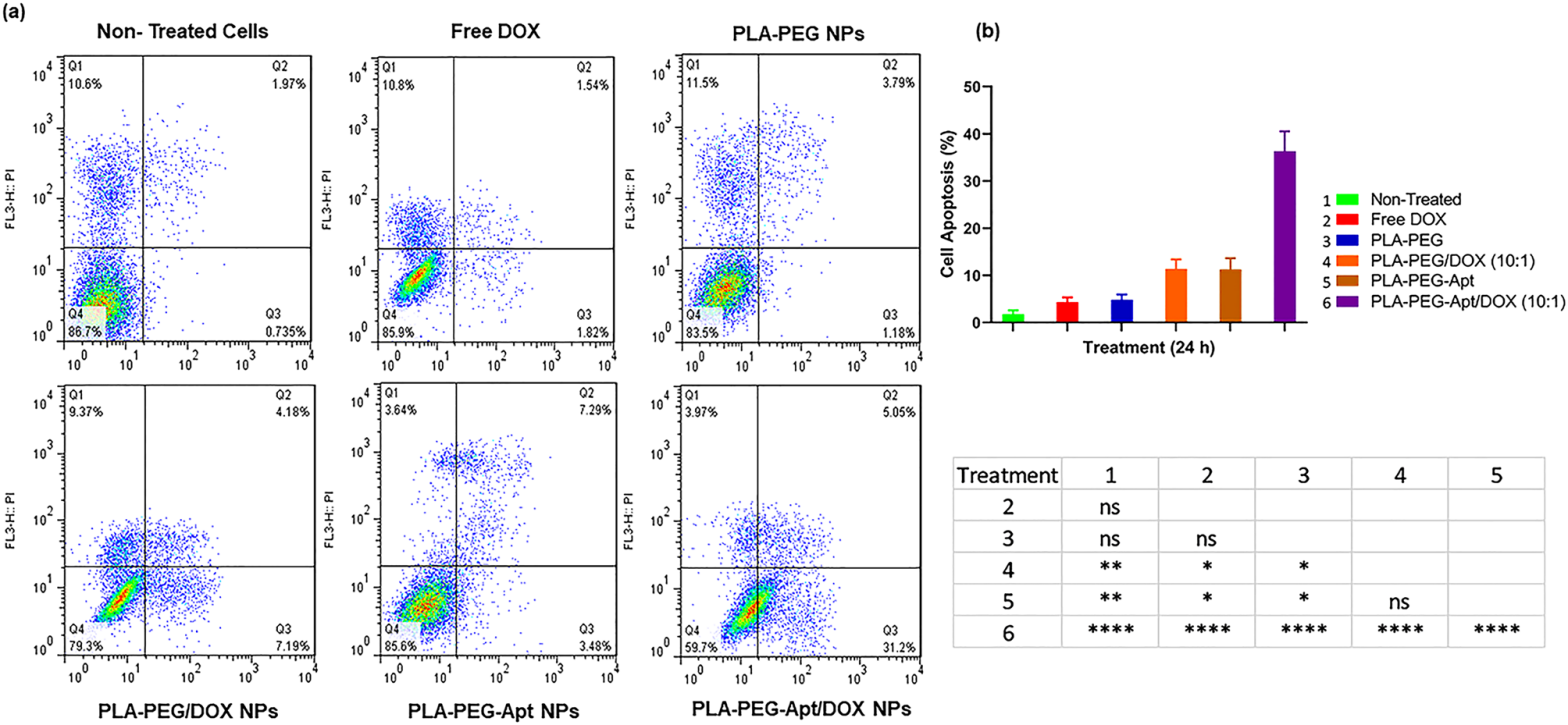
Cellular apoptosis. (**a**) Flow cytometry histograms of A-549 cells treated with Free DOX, PLA-PEG NPs, PLA-PEG/DOX NPs, PLA-PEG-Apt NPs, and PLA-PEG-Apt/DOX NPs for 24 h using Annexin V-FITC/PI staining (Q1: dead cells with Annexin V-FITC (−)/PI (+), Q2: late apoptotic cells with Annexin V-FITC (+)/PI (+) Q3: live cells with AnnexinV-FITC (−)/PI (−), Q4: early apoptotic cells with Annexin V-FITC (+)/PI (−)). (**b**) The number of apoptosis cells was counted with a FACS Calibur flow cytometer for A-549 cells (P-value: ns is not-significance (≥ 0.05). * is significant between 0.01 and 0.05., ** is significant between 0.001 and 0.01., and ****, significant < 0.0001).

The percentage of apoptosis induction in various formulations such as free DOX, PLA-PEG NPs, PLA-PEG/DOX NPs (w/w ratio 10:1), PLA-PEG-Apt NPs, and PLA-PEG-Apt/DOX NPs (w/w ratio 10:1) was evaluated in A-549 cells for 24 h by flow cytometry analysis of Annexin V-FITC (Fig. 7). As shown in Fig. 7a, the percentage of apoptosis for A-549 cells treated with free DOX and PLA-PEG NPs was 4.35 ± 0.81% and 4.82 ± 0.94% within 24 h, respectively, which showed the lowest apoptosis. Whereas, the percentage of cell apoptosis of PLA-PEG/DOX NPs (w/w ratio 10:1) and PLA-PEG-Apt NPs increased to 11.37 ± 1.65% and 11.25 ± 1.94% after 24 h, respectively. However, PLA-PEG-Apt/DOX NPs at w/w ratio 10:1 with 36.3 ± 3.44% apoptosis showed considerably higher percentage of dead cells than PLA-PEG/DOX NPs and PLA-PEG-Apt NPs in A-549 cells for 24 h. As indicated in Fig. 7b, there were statistically significant differences with P < 0.0001 between PLA-PEG-Apt/DOX NPs at w/w ratio 10:1 and other formulations. The results indicated that PLA-PEG-Apt/DOX NPs at w/w ratio 10:1 induced significantly higher apoptosis rate compared to non-targeted form and other formulations on A-549 cancer cells. This is due to the reason that MUC1 aptamer enhanced selective targeted binding to the MUC1 receptor which is overexpressed on A-549 cancer cells leading to higher cellular uptake of PLA-PEG-Apt/DOX NPs into A-549 cells through MUC1 receptor-mediated endocytosis and subsequently induced and increased apoptosis^58^.

**Figure 8:**
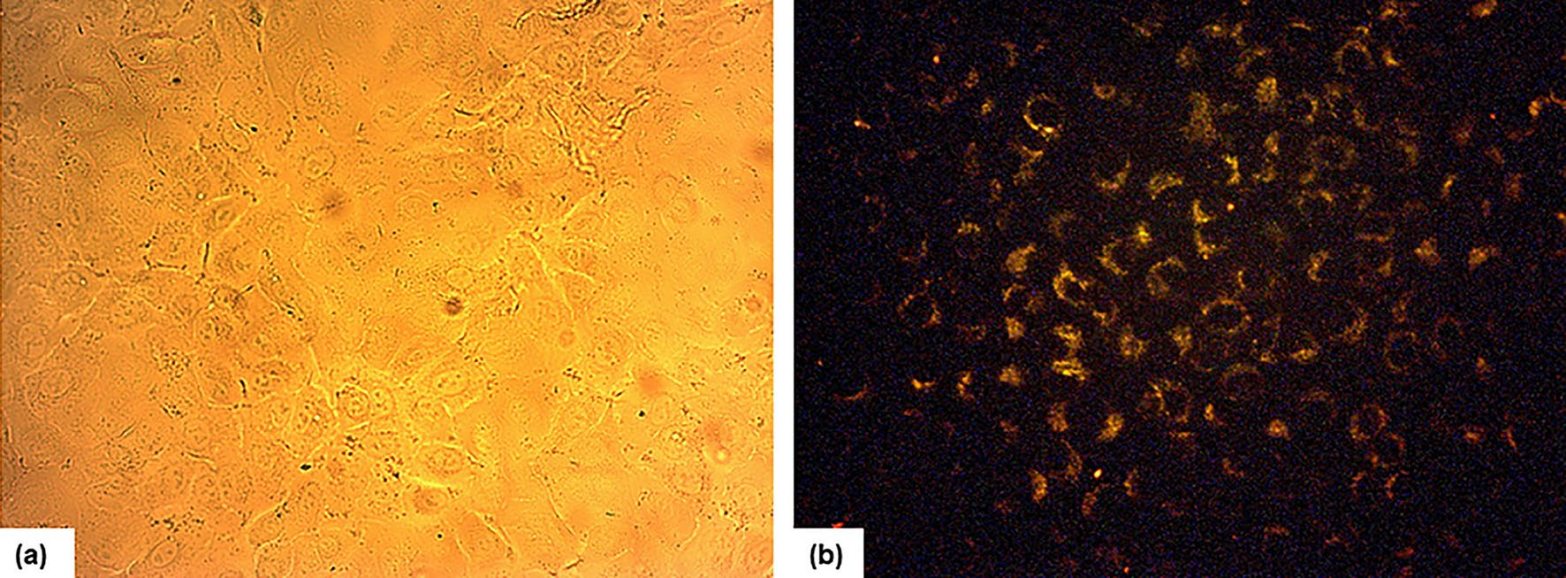
Fluorescence imaging of PLA-PEG-Apt/DOX NPs in A-549 cells. (**a**) Bright field, and (**b**) Dark field for A-549 cells.

Furthermore, fluorescence microscopy imaging (Fig. 8) also confirm this issue. The internalization and localization of PLA-PEG-Apt/DOX NPs at w/w ratio 10:1 in A-549 cells were examined by fluorescence microscopy. As displayed in Fig. 8, enhanced fluorescence signals were observed from PLA-PEG-Apt/DOX NPs at w/w ratio 10:1 in A-549 cells which demonstrating a significant uptake of nanoparticles in A-549 cancer cells and the image clearly indicated the internalization and accumulation of PLA-PEG-Apt/DOX NPs in A-549 cancer cells. The result suggesting again that binding aptamer to the MUC1 receptor facilitated the uptake of prepared nanoparticles into the cells^48,58,59^. Therefore, PLA-PEG-Apt/DOX NPs at w/w ratio 10:1 acted as potential nanoformulation for MUC1 positive cancer cells treatment.

## Conclusions

In this work, we prepared PLA-PEG-Apt/DOX NPs at w/w ratio 10:1 (PLA-PEG: DOX) by double emulsion method and evaluated their antitumor efficacy in MUC1 protein overexpressed A-549 cells. The results demonstrated that PLA-PEG-Apt/DOX NPs at w/w ratio 10:1 with desired particle size and high surface charge showed 57% controlled release of the total drug during 96 h. Furthermore, PLA-PEG-Apt/DOX NPs at w/w ratio 10:1 with 36.3 ± 3.44% apoptosis after 24 h showed enhanced DOX delivery to A-549 cancer cells. Therefore, MUC1 aptamer led to higher cellular uptake of PLA-PEG-Apt/DOX NPs into A-549 cells and increased apoptosis. Taken together, PLA-PEG-Apt/DOX NPs can be used as targeted drug delivery system for efficient anticancer drug delivery to MUC1 positive cancer cells.

## Supplementary Information

Supplementary Information 1.

Supplementary Information 2.

Supplementary Information 3.
