# Supplementary figures and images for: Targeting lung cancer cells with MUC1 aptamer-functionalized PLA-PEG nanocarriers
Source: Sci Rep. 2022 Mar 18;12:4718. doi: 10.1038/s41598-022-08759-z (PMC8933396; doi:10.1038/s41598-022-08759-z)

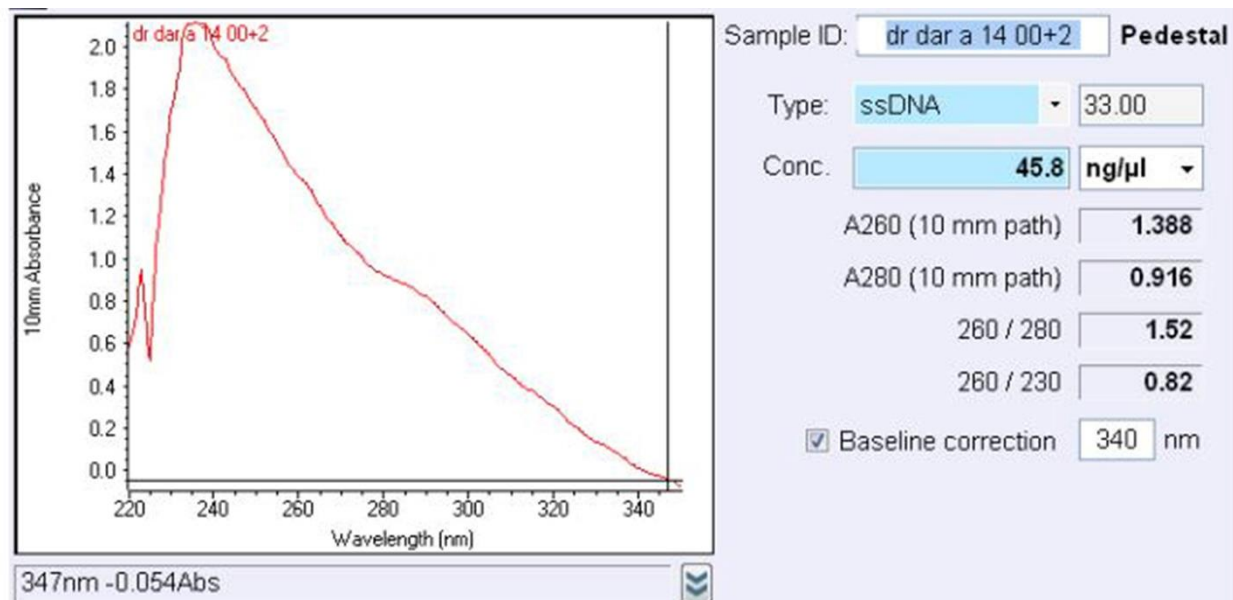

*Supplementary information for comment 1 of the reviewer 2: capping ratio of the aptamer*

Supplement: Supplementary file 1 — Supplementary Information 1. [file 41598_2022_8759_MOESM1_ESM.pdf]
