# Supplementary material for: Targeting lung cancer cells with MUC1 aptamer-functionalized PLA-PEG nanocarriers
Source: Sci Rep. 2022 Mar 18;12:4718. doi: 10.1038/s41598-022-08759-z (PMC8933396; doi:10.1038/s41598-022-08759-z)
Supplement: Supplementary file 2 — Supplementary Information 2. [file 41598_2022_8759_MOESM2_ESM.pdf]

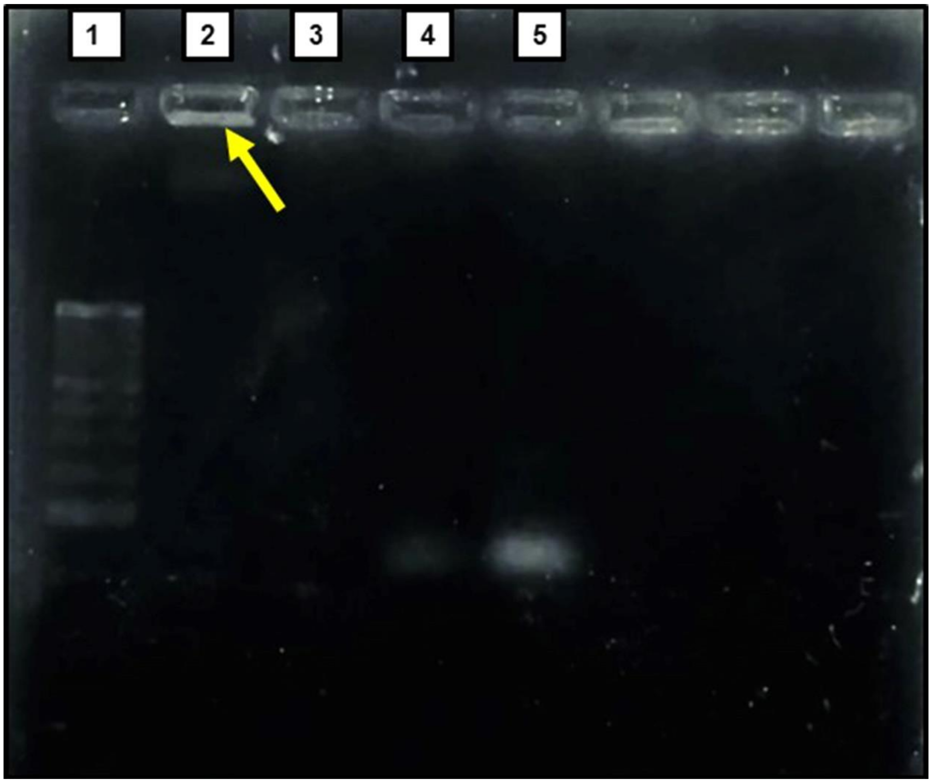

Supplementary for Figure 4. Confirmation of NPs-aptamer conjugation (uncropped gel). 1: ladder(100bp), 2: PLA-PEG-Apt/DOX NPs, 3: PLA-PEG, 4: mixture of aptamers and PLA-PEG, 5: free aptamer (Apt).
