# Supplementary material for: Targeting lung cancer cells with MUC1 aptamer-functionalized PLA-PEG nanocarriers
Source: Sci Rep. 2022 Mar 18;12:4718. doi: 10.1038/s41598-022-08759-z (PMC8933396; doi:10.1038/s41598-022-08759-z)
Supplement: Supplementary file 3 — Supplementary Information 3. [file 41598_2022_8759_MOESM3_ESM.pdf]

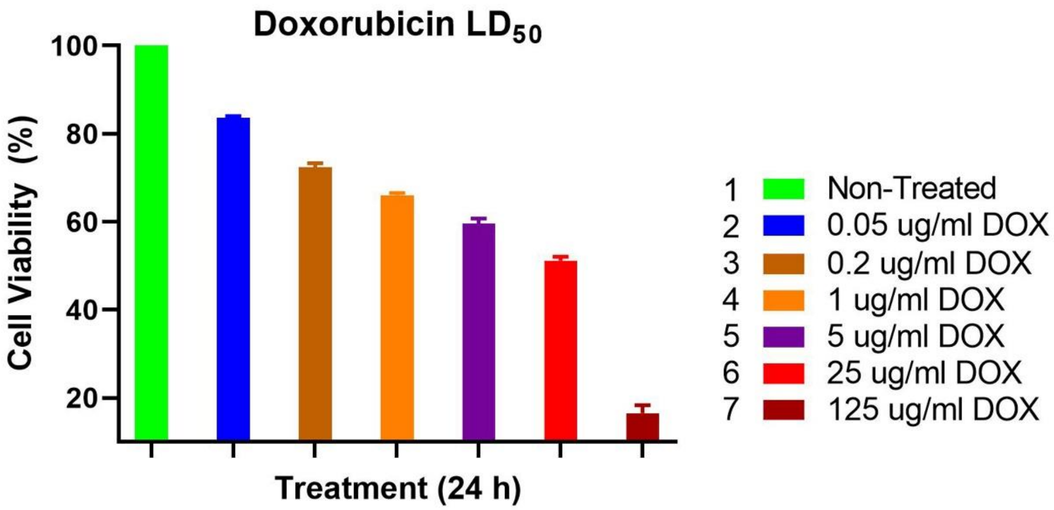

Supplementary information for comment 3 of the reviewer 2: Dose-dependent cytotoxicity and IC50 of Doxorubicin
